# A cell adhesion-promoting multi-network 3D printing bio-ink based on natural polysaccharide hydrogel

**DOI:** 10.3389/fbioe.2022.1070566

**Published:** 2022-11-28

**Authors:** Yong Qi, Shuyun Zhang, Yanni He, Shuanji Ou, Yang Yang, Yudun Qu, Jiaxuan Li, Wanmin Lian, Guitao Li, Junzhang Tian, Changpeng Xu

**Affiliations:** ^1^ Department of Orthopaedics, Guangdong Second Provincial General Hospital, Guangzhou, China; ^2^ Guangdong Second Provincial General Hospital, Postdoctoral Research Station of Basic Medicine, School of Medicine, Jinan University, Guangzhou, China; ^3^ Department of Ultrasound, Institute of Ultrasound in Musculoskeletal Sports Medicine, Guangdong Second Provincial General Hospital, Guangzhou, China; ^4^ Department of Medical Information, Guangdong Second Provincial General Hospital, Guangzhou, China; ^5^ Department of Medical Iconography, Guangdong Second Provincial General Hospital, Guangzhou, China

**Keywords:** 3D printing, bio-ink, tissue engineering, multi-network hydrogel, gellan gum

## Abstract

Due to its high biosafety, gellan gum (GG) hydrogel, a naturally occurring polysaccharide released by microorganisms, is frequently utilized in food and pharmaceuticals. In recent years, like GG, natural polysaccharide-based hydrogels have become increasingly popular in 3D-printed biomedical engineering because of their simplicity of processing, considerable shear thinning characteristic, and minimal pH dependence. To mitigate the negative effects of the GG’s high biological inertia, poor cell adhesion, single cross-linked network, and high brittleness. Mesoporous silica nanospheres (MMSN) and Aldehyde-based methacrylated hyaluronic acid (AHAMA) were combined to sulfhydrated GG (TGG) to create a multi-network AHAMA/TGG/MMSN hydrogel in this study. For this composite hydrogel system, the multi-component offers several crosslinking networks: the double bond in AHAMA can be photocrosslinked by activating the photoinitiator, aldehyde groups on its side chain can create Schiff base bonds with MMSN, while TGG can self-curing at room temperature. The AHAMA/TGG/MMSN hydrogel, with a mass ratio of 2:6:1, exhibits good cell adhesion, high strength and elasticity, and great printability. We believe that this innovative multi-network hydrogel has potential uses in tissue regeneration and biomedical engineering.

## Introduction

For regenerative medicine, pharmacokinetics, and other biological studies, 3D bioprinting enables for fine control of material shape while depositing living cells, growth factors, and other things to simulate real tissue structures. Due to its capacity to swiftly and cheaply customize particular medical items to patients, 3D bioprinting has a lot of potential in the fields of tissue engineering and medical devices (Mironov et al., 2009; Norotte et al., 2009). One of the most challenging problems in 3D printing for biomedical engineering is the creation of bio-inks (Panwar and Tan, 2016). From the perspective of cytocompatibility, an ideal bio-ink should satisfy the biological requirements of offering a favorable environment for cell adhesion and growth (Roth et al., 2004; Wallace et al., 2014). Bio-inks should have the physical characteristics of a gel, meaning they should have a specified viscosity and preserve continuity when extruded or poured (Gu et al., 2016). However, excessive viscosity can cause cell death and gel breakup since it increases the shear force needed for extrusion (Kong et al., 2003). In terms of mechanical properties, the extruded gel needs to be solid and rigid enough to maintain the structural integrity and designability of the printed material (Frampton et al., 2011). The hydrogel can be functionally changed and has tunable strength and degradability. Functionalized hydrogels can be cross-linked chemically or physically to transform from a liquid to a solid state (Luo et al., 2022). Some of them respond to particular temperatures, pH levels, and light wavelengths and can cure quickly in the appropriate environments to produce 3D printing inks (Dong et al., 2022; Liu et al., 2022). In addition, hydrogels exhibit mechanical properties that are similar to those of tissue and a network structure resembling the extracellular matrix. Due to its highly hydrated network structure, it has tremendous potential for biocompatibility and enables for gas exchange and metabolic transfer of nutrients or waste products (Zhang et al., 2021; Zhu et al., 2022). Because of these qualities, hydrogels are a commonly used material for tissue engineering and are utilized for both drug delivery and two- or three-dimensional tissue engineering scaffolds. Because they satisfy the physical and biological demands of bio-inks, hydrogels function synergistically and are crucial components of 3D printing materials.

Typically, there are three types of hydrogel inks in common use, the first being natural polymers. e.g., alginate, agar, gelatin, cellulose, hyaluronic acid, silk protein, and collagen. Their advantage is good biocompatibility, but they must be adjusted to accomplish rapid healing because of their weakness and difficulty in molding (Oyen, 2014; Sharma and Tiwari, 2020). Hydrogels made of synthetic polymers are the second kind of substance. These materials’ benefits include good strength, ease of processing and shaping, and strength. They are brittle, do not match the mechanical characteristics of the surrounding tissue, have poor cytocompatibility, and frequently require compounding with biologically active materials or grafting with biologically active functional groups, as well as the inclusion of growth hormones (Ahmed, 2015). The third form of hydrogel bioink, known as composite hydrogels, has arisen to solve the drawbacks of these materials while retaining their beneficial features. Composite hydrogels are made of both natural and synthetic polymers(Zhang et al., 2011; Thoniyot et al., 2015).

As a polysaccharide hydrogel of natural microbial origin, gellan gum (GG) is widely used in the pharmaceutical and food industries because it is non-cytotoxic (Das and Giri, 2020). The thixotropic qualities of GG and its capacity to gel at room temperature make it a viable candidate for use in 3D bioprinting (Oliveira et al., 2010). However, GG’s poor mechanical characteristics, brittleness, and high dissolving temperature, which are unfavorable to cell loading and cellular inertia, have also impeded its advancement in biomedical engineering. It is practical to enhance the mechanical and cytocompatibility of GG by compounding it with a biologically active natural hydrogel (Silva-Correia et al., 2013; Balasubramanian et al., 2018). At the same time, GG’s solubility conditions can be improved by chemical alteration, making it more suitable for 3D bioprinting and water-soluble at room temperature (Douglas et al., 2014; Koivisto et al., 2019). Hyaluronic acid (HA) is a non-sulfated glycosaminoglycan found throughout the body, such as in the eye’s vitreous humor and the extracellular matrix (ECM) of cartilage tissue (Fraser et al., 1997). Its biological characteristics and structural characteristics enable it to facilitate cell signaling in tissues safely and reliably (Toole, 2004). Due to these benefits, HA and its derivatives have been used as medical treatments in clinical settings for more than 30 years (Kuo, 2005). But the poor mechanical qualities and Severe gelation conditions of HA as a 3D printing ink make it far less advantageous (Mironov et al., 2008). Normally, researchers chose to modify chemicals to increase their potential to cure (Skardal et al., 2010b), either by combining them with stronger polymers or functional nanoparticles to improve their mechanical characteristics and conformational capacities (Skardal et al., 2010a).

In synthesizing the above research background, in this study, GG was first mercapturized (TGG) in order to make it water soluble at room temperature (RT) and simple to combine with other substances. To further enhance TGG’s cytocompatibility, aldehyde methacrylate-modified HA (AHAMA) was blended in. TGG’s brittleness, on the other hand, balances AHAMA’s suppleness, making it a better choice as a 3D printing ink substance. In addition to TGG cooling curing, HAMA is abundant in radicals for initiator activation to enable photocrosslinking as a second gel curing method, improving the gel stability of multi-component hydrogel inks. Secondly, mesoporous silica nanoparticles (MSN) were selected as functionalized components in this study. The amino-modified mesoporous silica nanoparticles (MMSN) obtained by amination of MSN could be grafted onto AHAMA side chains by Schiff base reaction. The stability of MMSN in the hydrogel system is ensured by chemical grafting. While creating a third crosslinking network to produce AHAMA/TGG/MMSN hydrogels in multiple crosslinking networks, which prevents MMSN escape from causing cytotoxicity. Through testing, we have demonstrated that the AHAMA/TGG/MMSN hydrogel satisfies the needs of micron-scale 3D printing of bio-inks by having moderate and quick curing conditions, good mechanical properties, and considerable cell adhesion. We believe that this hydrogel can meet the requirements of various configurations, combine the needs of 3D printing bio-inks, and has a significant amount of potential for use in tissue engineering.

## Materials and method

### Materials

GG (BR, formula weight = 1,000 kg/mol), HA (BR, Mw = 750,000∼1,000,000), Methacrylate, Sodium Periodate, L-cysteine hydrochloride monohydrate, and Hexadecyltrimethylammonium bromide (CTAC) were purchased from Sigma-Aldrich. N-(3-Dimethylaminopropyl)-N′-ethylcarbodiimide hydrochloride (EDAC), N-Hydroxysuccinimide (NHS), and Triethanolamine (TEA), 3-Aminopropyltriethoxysilane (APTES) and Tetraethyl orthosilicate (TEOS) were of analytical grade and purchased from Qi Yun Biotechnology Co., Ltd. (China). All other reagents were commercially available, of analytical grade, and used without further purification.

### Preparation of TGG

The GG (2%, wt%) solution was prepared at 80°C, then cooled at RT. 100 ml of reaction solution consisting of 0.6 g 1-(3-dimethyl aminopropyl)-3-ethyl carbodiimide hydrochloride, 0.6 g L-cysteine hydrochloride, 0.6 g NHS and deionized (DI) water as solvent. The reaction system was adjusted to pH = 5.0 by using 0.1 M HCL and 0.1 M NaOH. Add the cooled GG gel to the above reaction working solution and pass nitrogen for 3 min. Protect from light, seal tightly and leave overnight at 4°C. After the reaction was completed, the gel was packed into dialysis bags (MWCO 14 kDa) and placed at 4°C, protected from light for 1 week, with daily changes of DI water. After 1 week, a small amount of silver nitrate solution was added to the dialysate sample, and if the solution remained turbid, the dialysis was complete. The sample is freeze-dried and stored at −20°C, sealed, and protected from light. The sample is named TGG.

### Synthesis and characterization of AHAMA

Dissolving 1 g HA in 100 ml DI water, add 5 ml 0.5 M sodium periodate, stir at 250 rpm for 2 h at RT, and protect from light. 1 ml of ethylene glycol was then added to deactivate the unreacted periodate. The samples were dialyzed using DD water for 3 days. The sample was removed from the completed dialysis, freeze-dried, and AHA was obtained. Afterward, dissolve 1 g AHA in 100 ml DI water and stir until completely dissolved. Add 1 ml of methacrylate and react for 12 h at pH = 8.0–8.5. The whole process is carried out on the ice to ensure a cryogenic environment. After the reaction, the solution was dialyzed for 2 days and freeze-dried to obtain AHAMA. 1H nuclear magnetic resonance (1H NMR, 500 MHz) spectra were recorded on an AVANCE III HD 600 NMR spectrometer (Bruker, Germany).

### Preparation of MMSN

As a mesoporous template, dissolve 2 g CTAC in 20 ml of DD water. Then 0.85 mLTEA was added and mixed at RT for 0.5 h. The reaction system was transferred to an oil bath at 80°C, and 0.75 ml TEOS was added dropwise. After 1 h of hydrolysis and condensation, the product was collected by centrifugation and washed several times with ethanol to remove the residual reaction products. The collected product was extracted with a methanolic solution containing 1 wt% sodium chloride (NaCl) for 3 h at RT to remove the template CTAC and washed and lyophilized to obtain the MSN. 50 mg of MSN was obtained and dispersed in 50 ml of ethanol, the solution was refluxed for 4 h, and then 100 µl of APTES was added. After centrifugation of the above working solution, the samples were collected and washed with DI water and then freeze-dried to obtain MMSN.

### Preparation and characterization of AHAMA/TGG/MMSN hydrogel

Prepare 2% (w/v) TGG solution at 80°C in a constant temperature water bath. Dissolve AHAMA in PBS (pH = 7.4) (6%, w/v) and add I-2959 initiator (0.2%, w/v) and MMSN (concentration gradients of 0.5, 1, 2 and 4%, w/v) then mix well. Mix the above two solutions in equal volumes, stir well quickly, then pour into the mold (*Φ* = 20 mm) and irradiate under UV light (365 nm, 50 mW/cm^2^) for 5 min to form AHAMA/TGG/MMSN composite hydrogel.

To observe the internal structure of the composite hydrogel and to account for changes in porosity, SEM was employed to characterize the hydrogel samples before and after the composite. Preparation of AHAMA, TGG, AHAMA/TGG, AHAMA/TGG/MMSN samples were kept at −80°C for 24 h before being freeze-dried. Then, the freeze-dried samples were carefully fractured and sputter-coated with gold for SEM observation. Statistical analysis of the pore size of different component hydrogels using ImageJ. FT-IR was employed to characterize the functional group changes during gel curing. The swelling rate of the hydrogels was measured by measuring the wet weight of the gels immersed in PBS for different lengths of time. Swelling (Qs, %) was expressed as follows: Where Ws is the sample’s swollen mass and Wd is its dry mass (Ye et al., 2013; Zhang et al., 2021).

$$Qs=\frac{Ws-Wd}{Wd}\times 100\%.$$ (1)

### Mechanical properties and print performance of AHAMA/TGG/MMSN hydrogel

The rotational rheometer demonstrates the change in hydrogels’ storage modulus (G′) and loss modulus (G′) under different conditions. Under 37°C; frequency: 1.0 Hz; strain: 1.0% conditions, the flow dynamic hydrogels were exposed under 50 mW/cm^2^ UV irradiation and the changes in G′ and G′ were recorded. The test frequency was varied to record changes in the modulus of different hydrogels. The same-size hydrogel samples were placed in a universal mechanics machine and set up for cyclic compression experiments at 80% to observe the recovery of the hydrogel.

Vary the test strain to obtain the hydrogel shear thinning ability and analyze its printability. A composite hydrogel with different components and different levels of MMSN is prepared, cured in a mold, and later photographed under an optical microscope. Analysis of the printability and stability of the composite hydrogels.

### Cell culture

After sterilization by Co60 radiation, the AHAMA, TGG, AHAMA/TGG, and AHAMA/TGG/MMSN were placed in 24 well-sterile orifice plates. The hydrogel materials used in the cell experiments were all freeze-dried. All BMSCs (third generation, from ATCC) were cultured with complete BMSC medium comprising 89% α-MEM basic medium (Gibco, United States), 10% Fetal Bovine Serum (Gibco, United States), and 1% Penicillin-Streptomycin (BI, United States, 10,000 U/ml∼10 mg/ml) and were seeded with a density of 1 × 10^4^ cells per well. Four repetitions were performed. In addition, all cell-handling procedures were performed in a sterile laminar flow hood, and all cell-culture incubation steps were conducted at 37°C with 5% CO_2_.

### Cell proliferation and adhesion

After the inoculation of cells, AHAMA, TGG, AHAMA/TGG, and AHAMA/TGG/MMSN were washed with PBS (pH = 7.4) on the day 1, 3, and 5, respectively. The CCK-8 and AO/EB kit were adopted to test the number of cells remaining on the template to obtain cell proliferation. In contrast, immunofluorescence testing was adopted to detect the secretion of vinculin. Specifically, AHAMA, TGG, AHAMA/TGG, and AHAMA/TGG/MMSN samples cultured for 3 days were fixed with 4% paraformaldehyde (15 min), permeabilized in 0.1% Triton X-100 (5 min) and blocked with 1% bovine serum albumin (BSA) for 30 min at RT. Samples were incubated with an anti-vinculin antibody (1/250 in PBS + 1% BSA) for 12 h. Alexa Fluor^®^ 488 (1/500 in PBS with 1% BSA) was used as the secondary antibody. DAPI (4,6-diamidino-2-phenylindole) was used for the nucleus (in blue). Immunofluorescence photographs were taken with confocal laser scanning microscopy (LSM880, Carl Zeiss Micro-Imaging GmbH, Jena, Germany). To quantify vinculin levels in cell fluorescence, images were analyzed by ImageJ following the previously mentioned protocol (Zhang et al., 2021). Select single-channel vinculin fluorescence images, then adjust the threshold. Three thresholds of the same sample were selected for measurement, each containing 5 cells. The software will then calculate the mean gray value by using the default algorithm.

### Statistical analysis

All statistical analyses were performed using analysis of variance (one way ANOVA) and Tukey’s test, in which differences were considered significant for ns = *p* > 0.05/*p < 0.05/**p < 0.01/***p < 0.001.

## Results and discussion

### Characterization of AHAMA/TGG/MMSN hydrogel

**FIGURE 1:**
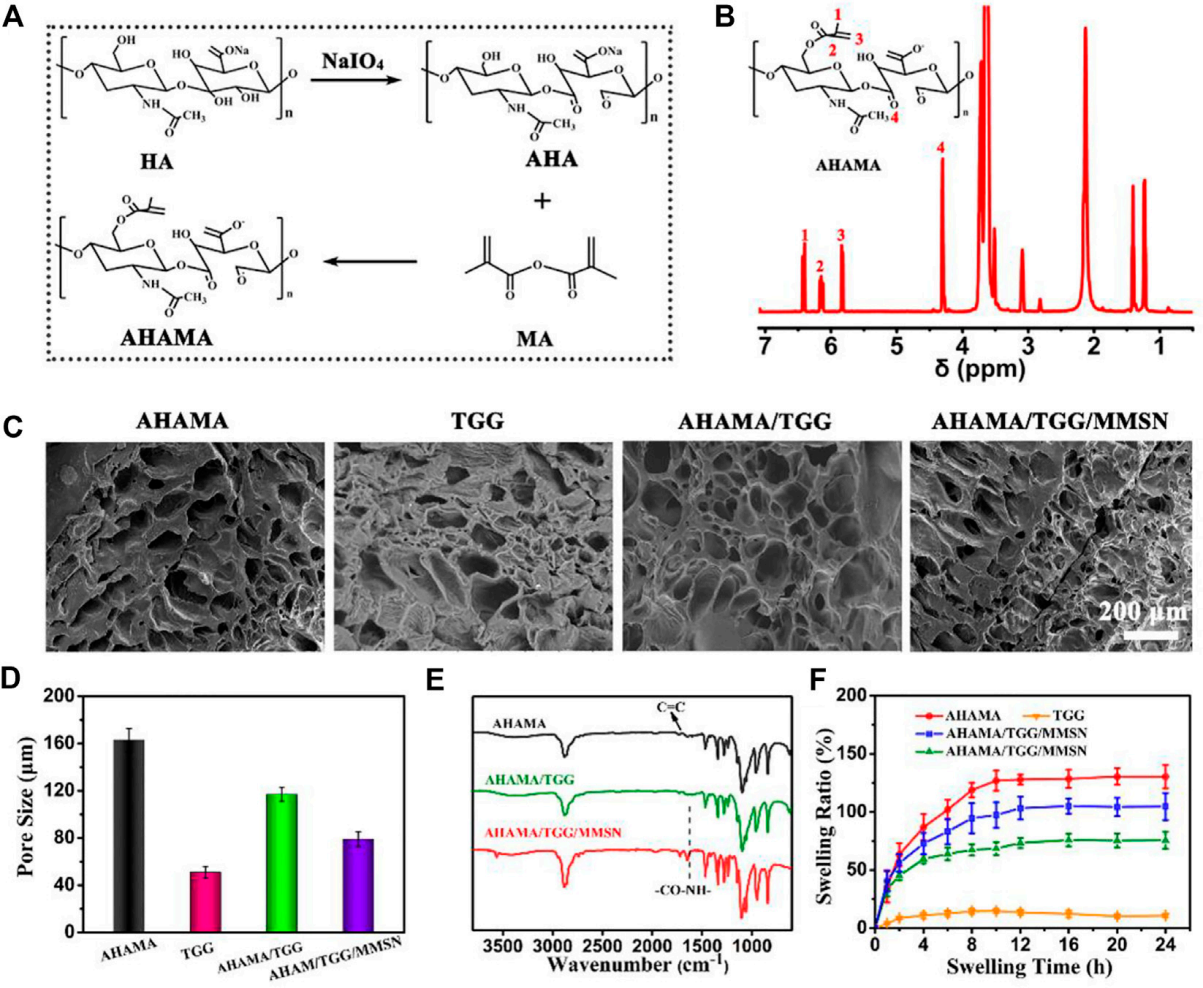
**(A)** Chemical synthesis of AHAMA conjugate by a two-step approach. **(B)** 1H-NMR analysis of AHAMA conjugate. **(C)** SEM images of AHAMA, TGG, AHAMA/TGG and AHAMA/TGG/MMSN hydrogels, and **(D)** their corresponding pore diameter. **(E)** FT-IR spectra of the prepared AHAMA, AHAMA/TGG and AHAMA/TGG/MMSN scaffolds. **(F)** Equilibrium swelling rate of the different hydrogels at 24 h.

It is confirmed that maleic anhydride was successfully grafted onto oxidized HA by the presence of H at positions 1, 2, and three of AHAMA in the NMR pattern, as illustrated in Figures 1A,B. By combining the TGG and MMSN components, the hydrogel’s FT-IR profile (Figure 1E) was also changed. The Schiff base reaction between the aminated MMSN and AHAMA was confirmed by the substitution of C = C for CO-NH in AHAMA. The interior structure of the different component hydrogels can be seen in the SEM diagram (Figure 1C), with TGG having smaller holes than AHAMA and displaying a larger hole structure. Following lamination, the AHAMA/TGG and AHAMA/TGG/MMSN hydrogels had larger pores than those of TGG. The statistical analysis results support the above findings, showing that AHAMA/TGG pores are larger than AHAMA/TGG/MMSN pores. We attribute this to the MMSN crowding out the pore space. The change in pore size will directly impact the hydrogel’s ability to swell. Because of its small pore size, the swelling statistics demonstrate that pure TGG hydrogel has inferior swelling capabilities. This is one of the factors leading to TGG’s apparent biological inertness. The swelling ratio of the AHAMA/TGG/MMSN hydrogel is substantially higher than that of TGG. With the addition of AHAMA, there was an increase in the number of pores, Schiff bases between MMSN and AHAMA formed, nanoparticles crowded the pore space, and AHAMA/TGG/MMSN solubility and pore size slightly decreased. The good swelling properties determine the material’s ability to retain water, interact with a liquid environment, and determine its biocompatibility. We found that compounding AHAMA/MMSN into the TGG system can produce multi-network cross-linking and enhance swelling through swelling ratio testing.

### Mechanical properties of AHAMA/TGG/MMSN hydrogel

**FIGURE 2:**
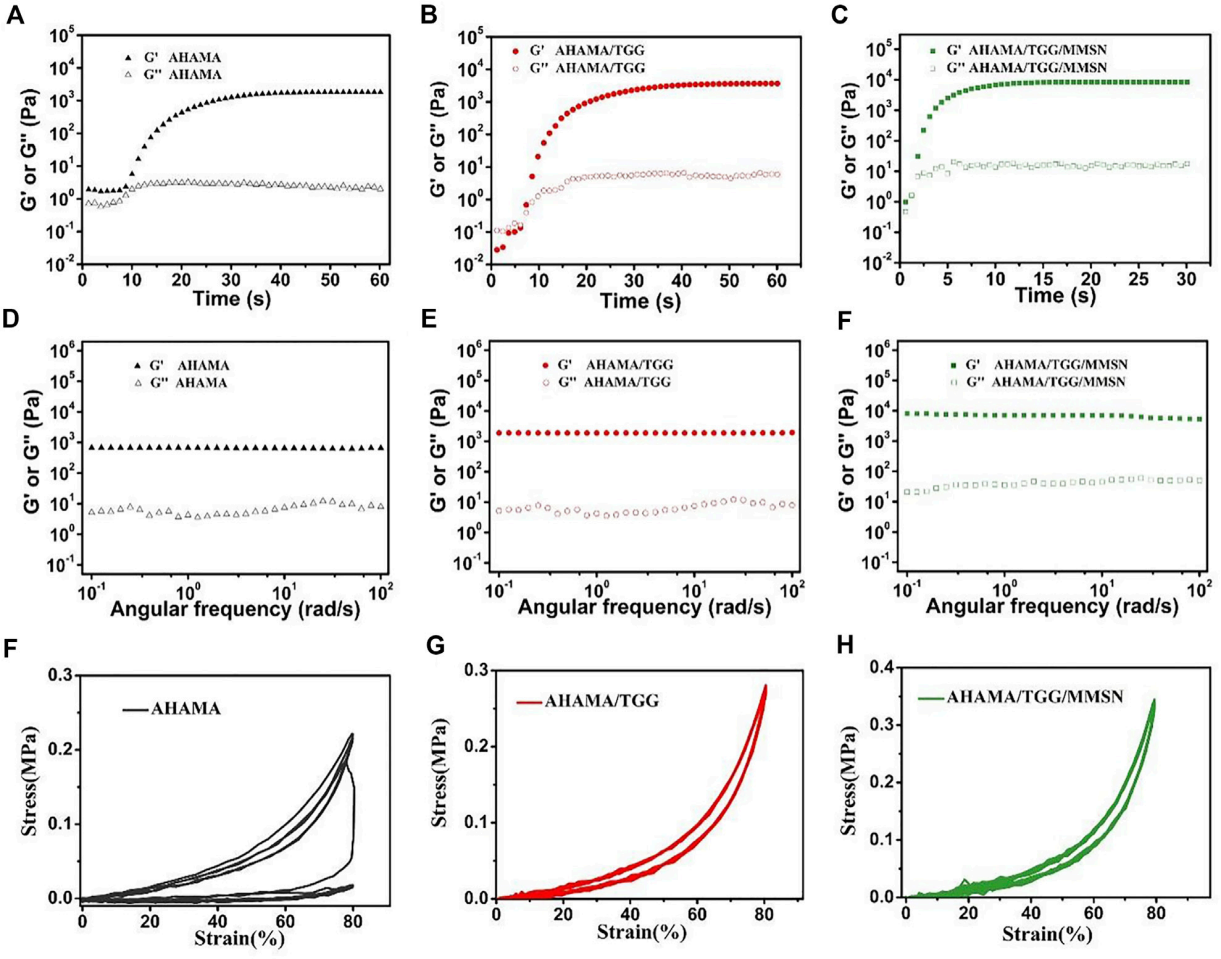
Gelation properties of AHAMA **(A)** AHAMA/TGG **(B)** and AHAMA/TGG/MMSN **(C)** hydrogel system using time sweeping of G′ and G″ under 50 mW/cm2 UV irradiation, and the correspondin) frequency-dependent at a strain of 1% **(D–F)** Twenty successive compressive loading-unloading cycles for the AHAMA **(F)** AHAMA/TGG **(G)** and AHAMA/TGG/MMSN **(H)** hydrogels with a strain of 80%.

As the curing time increases, G′ and G″ gradually intersect, suggesting that the state of hydrogel was between solid and fluid near this critical point (Wei et al., 2015). The curing time of AHAMA/TGG/MMSN is shorter than that of the other samples, reaching the gelation point in 3 s, as shown in Figures 2A–C. The composite hydrogels are more appropriate as 3D printing ink due to their quick curing qualities. As rotation frequency increased, the cured AHAMA/TGG/MMSN hydrogel’s G′ and G′ remained steady and were noticeably greater than those of AHAMA and AHAMA/TGG. TGG maintains its strength in the composite system, and the addition of MMSN increases the hydrogel’s strength even more to give the system a high level of stability and support for printing in various shapes. After curing, the mechanical characteristics of the hydrogel can be confirmed through cyclic compression tests. With a maximum stress intensity of only 0.24 MPa after the third cycle, it is evident that pure AHAMA collapses. When the hydrogel reaches a strain of 80%, compounding with TGG improves the hydrogel’s recovery capability while, to some extent, enhancing the maximum stress strength of the material. The addition of MMSN increases the AHAMA/TGG/MMSN hydrogel’s stability and provides it with a 0.36 MPa maximum compressive strength. In earlier experiments, naturally derived polymer-based 3D-printed hydrogels frequently failed to reach such great mechanical strength (Ye et al., 2018; Zhang et al., 2021).

### Print performance of AHAMA/TGG/MMSN hydrogel

**FIGURE 3:**
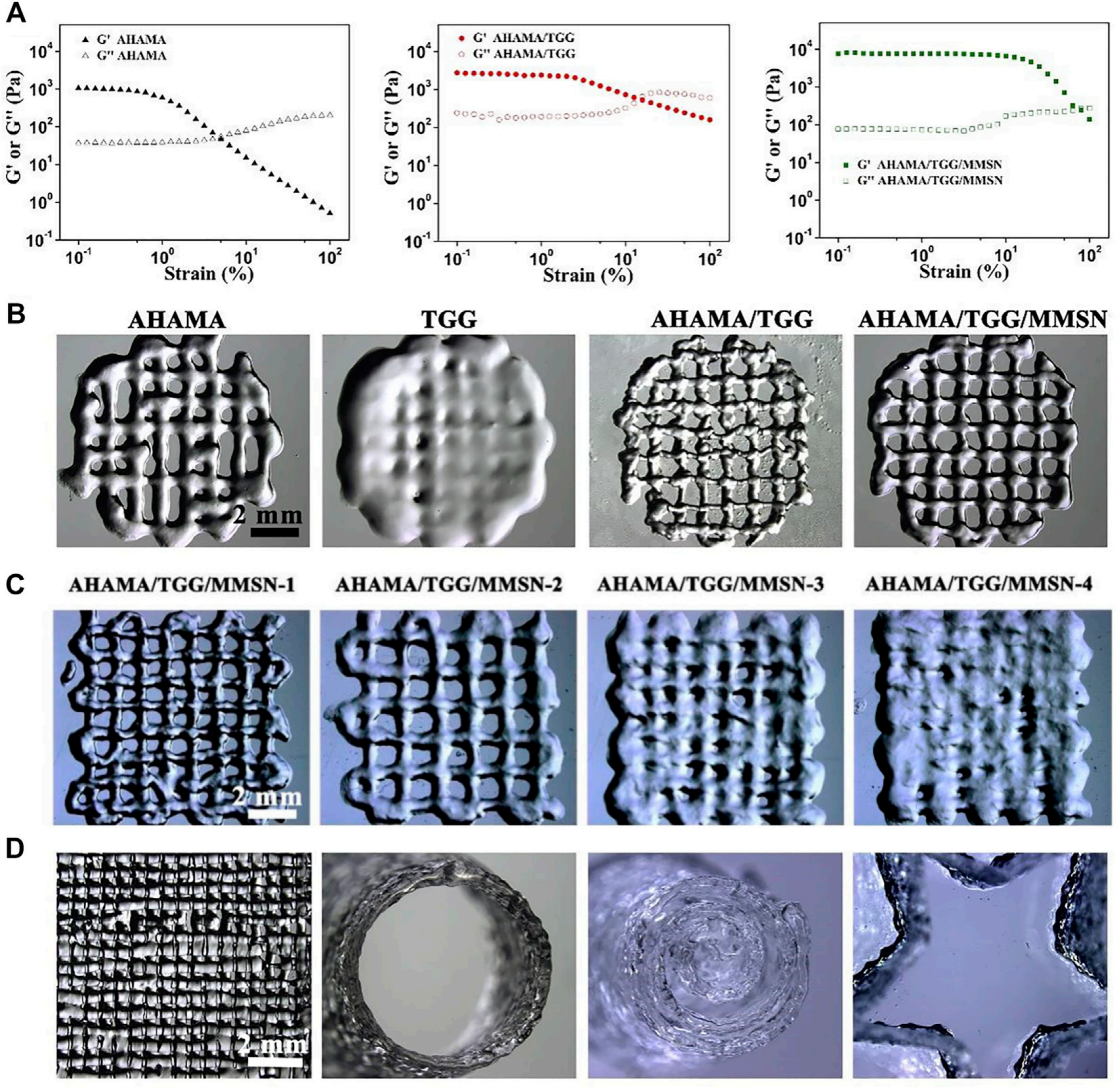
**(A)** Results of strain-dependent (w = 6.28 rad/s) oscillatory shear of AHAMA, AHAMA/TGG and AHAMA/TGG/MMSN hydrogels. **(B)** Printability of AHAMA/TGG and AHAMA/TGG/MMSN hydrogels. **(C)** Postural microscope images of AHAMA/TGG/MMSN hydrogel with different concentrations of MMSN 3D printing. **(D)** Images of various structure manufactured by 3D printed hydrogel.

To illustrate their printability and print parameter settings, we investigated the viscosity variations of bio-hydrogel inks under shear stress. For all component gel samples, G′ decreased as the shear frequency progressively rose. However, the relatively constant modulus of AHAMA/TGG/MMSN makes it easier for a continuous gel to develop during extrusion. The decrease in storage modulus indicates the ability of the semi-fluid or fluid to be extruded under shear to form a continuous material, which is necessary for extrusion printing. In this test, we can find that AHAMA, AHAMA/TGG and AHAMA/TGG/MMSN all have good shear thinning capability for extrusion printing. At the same time, we found that the AHAMA/TGG/MMSN group hydrogels were able to maintain a high modulus when shear forces were applied, which provided a mechanical basis for the construction of structurally stable three-dimensional scaffolds. While AHAMA, TGG, and AHAAMA/TGG are less precise and less printable, AHAMA/TGG/MMSN can print more precisely and has a diameter of 500 ± 35 μm. The printability of the composite hydrogel dramatically declines as MMSN concentration rises, with 1% (w/v) MMSN (sample AHAMA/TGG/MMSN-2) being found to be the ideal concentration by Figure 3C. Modeling was then used to confirm that when the MMSN content was 1%, the printed 3D conformational characteristics of the composite hydrogel were more stable in Figure 3D. A lattice-like print path is first set up using extrusion print curing to obtain a lattice-like hydrogel common to 3D printed tissue engineering scaffolds. The second image in Figure 3D shows a side view of the first grid-like support, where they show the printing of a single layer up to a thickness of 1 mm. The third image, showing the results of a multi-layer print, confirms that this hydrogel bio-ink remains stable when printed in multiple layers. The fourth diagram shows the printability of the hydrogel, i.e., it can be designed as a scaffold material in a variety of forms. Combining the information above, we came to the conclusion that the hydrogel may be used as a bio-ink for 3D printing when the composite mass ratio was AHAMA/TGG/MMSN = 2/6/1.

### Biocompatibility of AHAMA/TGG/MMSN hydrogel

**FIGURE 4:**
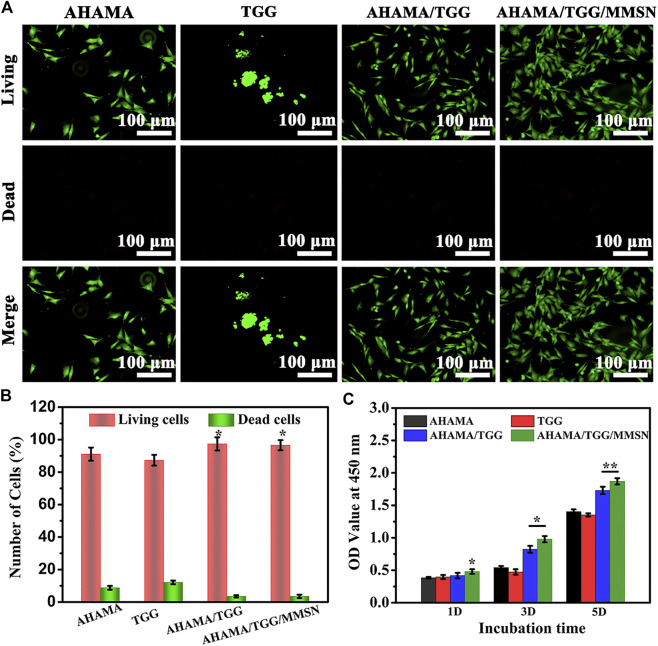
**(A)** Live/dead fluorescent images of BMSCs cells cultured on different scaffolds and **(B)** its quantitative analysis of live/dead cells on day 3. **(C)** Cell viability after contact with the 3D printing AHAMA, TGG, AHAMA/TGG and AHAMA/TGG/MMSN scaffolds.

The results of the live-dead staining (Figure 4) of the cells show that the hydrogels AHAMA, AHAMA/TGG, and AHAMA/TGG/MMSN are cytocompatible. On day 3, the cells in all of the aforementioned hydrogels seemed to be spreading, but the TGG cells were noticeably clumped together. It is well known that cell pseudopod dispersion enhances cell-environment interactions whereas cell clumping inhibits cell proliferation (Luo et al., 2022). The results of AO/EB staining are supplemented by the statistical analysis of living and dead cells, which shows that the AHAMA/TGG and AHAMA/TGG/MMSN hydrogels contain significantly more cells than the other groups (*p* < 0.05) and that pure TGG contains significantly more dead cells. As a result of the findings, it was shown that the AHAMA/TGG/MMSN group could considerably encourage cell growth (*p* < 0.01). (*p* 0.01). Although TGG still exhibits significant cellular inertia, we think that the addition of AHAMA and MMSN significantly improves TGG’s cytocompatibility.

### Cell adhesion

**FIGURE 5:**
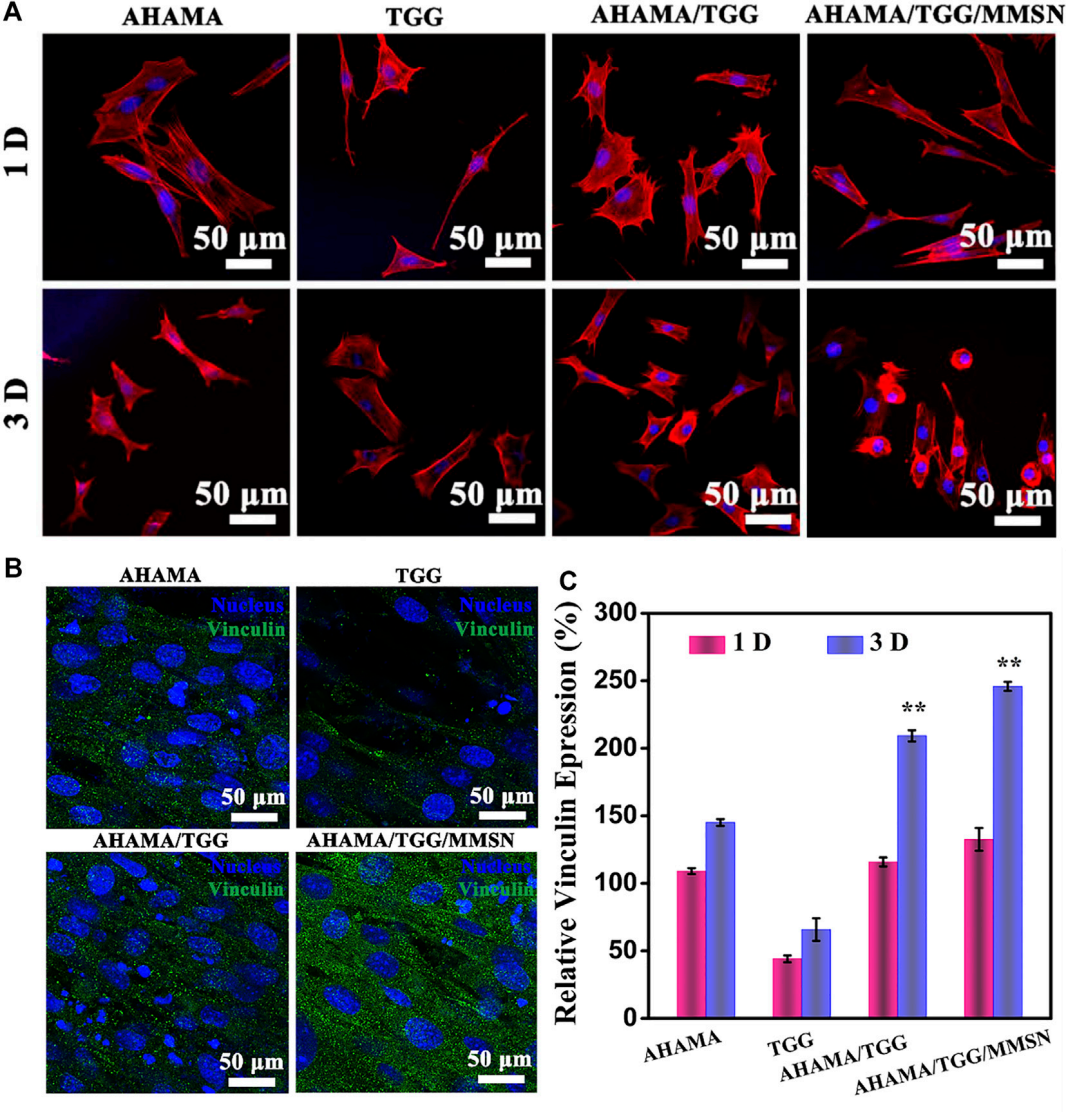
**(A)** CLSM images of stained BMSCs cells showing morphology adhered on the 3D printing AHAMA, TGG, AHAMA/TGG and AHAMA/TGG/MMSN scaffolds on day 1 and 3, respectively. **(B)** Fluorescence staining of nucleus (blue) and vinculin (green) of BMSCs cultured with various 3D printed scaffolds for 3 days: and **(C)** its corresponding quantitative results of expression of vinculin protein.

At one and a half days, immunofluorescence profiles of the cytoskeleton revealed cell spreading on the surface of several hydrogel components. The Figure 5A The poor status of cell spreading in TGG provides evidence of the hydrogel’s cellular inertness. The skeleton expanded more widely in the AHAMA and AHAMA/TGG/MMSN groups, allowing the cells to interact with their surroundings more effectively. Figure 5B shows that the secretion of vinculin was considerable and evenly distributed in the AHAMA/TGG/MMSN group, although it was significantly lower in the TGG samples compared to the other groups. This result was supported by the quantitative analysis that came after. The AHMA group’s vinculin secretion increased with time but did not differ significantly from the first day’s levels. Over the course of 3 days, the AHAMA/TGG composite hydrogel boosted vinculin secretion; this effect was amplified by the addition of MMSN. We propose that the three-component multi-network hydrogel can effectively promote cell adhesion.

## Conclusion

In this study, we successfully created a bio-hydrogel ink that can be printed in three dimensions using a variety of cross-linked natural polymeric ingredients. TGG and MMSN are used in this hydrogel system to supplement the challenging mechanical and print-curing conditions of AHAMA. At the same time, AHAMA completes TGG’s cytocompatibility and elasticity. The AHAMA/TGG/MMSN hydrogel exhibits good printability with a print accuracy of 500 μm, achieves 0.36 MPa in mechanical properties, and has good resilience properties. Additionally, the AHAMA/TGG/MMSN hydrogel demonstrates excellent cell attachment and good cytocompatibility. While the MMSN loaded in this system has additional research value in terms of drug administration, we think that this multi-network composite hydrogel has a high potential for further exploration of its capacity to be used as an implant material in the field of tissue repair.

## Data Availability

The original contributions presented in the study are included in the article/supplementary material, further inquiries can be directed to the corresponding authors.

## Nomenclature

Resource identification initiative: For this study we have no plan to take part in the RRID.
Life science identifiers: For this study we have no LSIDs.
