# Correction: A cell adhesion-promoting multi-network 3D printing bio-ink based on natural polysaccharide hydrogel

**DOI:** 10.3389/fbioe.2025.1636750

**Published:** 2025-06-12

**Authors:** Yong Qi, Shuyun Zhang, Yanni He, Shuanji Ou, Yang Yang, Yudun Qu, Jiaxuan Li, Wanmin Lian, Guitao Li, Junzhang Tian, Changpeng Xu

**Affiliations:** ^1^ Department of Orthopaedics, Guangdong Second Provincial General Hospital, Guangzhou, China; ^2^ Guangdong Second Provincial General Hospital, Postdoctoral Research Station of Basic Medicine, School of Medicine, Jinan University, Guangzhou, China; ^3^ Department of Ultrasound, Institute of Ultrasound in Musculoskeletal Sports Medicine, Guangdong Second Provincial General Hospital, Guangzhou, China; ^4^ Department of Medical Information, Guangdong Second Provincial General Hospital, Guangzhou, China; ^5^ Department of Medical Iconography, Guangdong Second Provincial General Hospital, Guangzhou, China

**Keywords:** 3D printing, bio-ink, tissue engineering, multi-network hydrogel, gellan gum

There was an error in the stereomicroscope image of the AHAMA/TGG/MMSN-4 group in Figure 3C as published. During the layout process of the manuscript, the figure is inadvertently overwritten by the image of the AHAMA/TGG/MMSN-3 group in Figure 3C. The corrected Figure 3C appears below.

**FIGURE 3:**
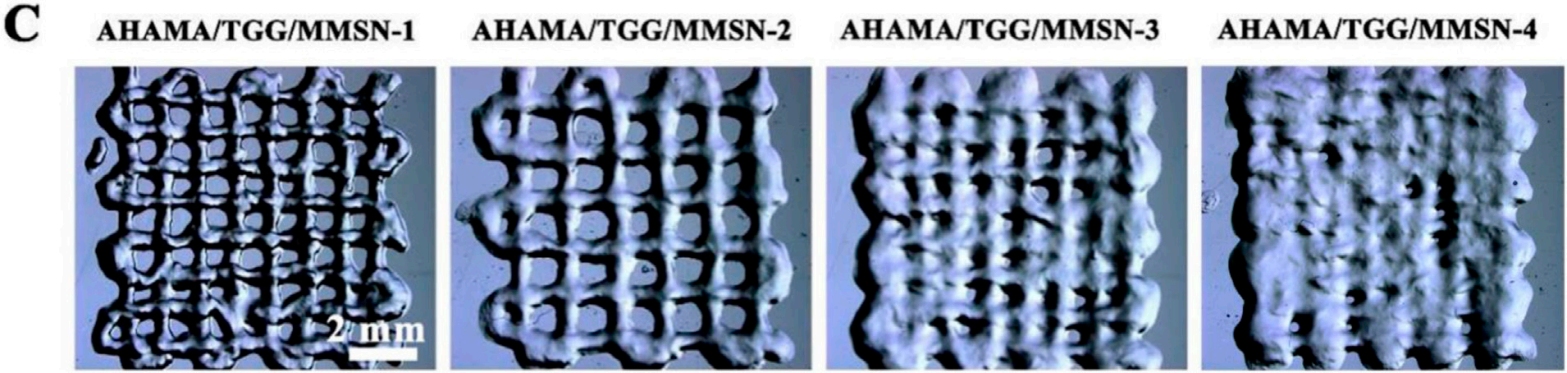
**(C)** Postural microscope images of AHAMA/TGG/MMSN hydrogel with different concentrations of MMSN 3D printing.

There was an error in the confocal laser scanning microscope (CLSM) image of the AHAMA/TGG/MMSN treatment group (3D) in Figure 5A as published. An incorrect image was used for Figure 5A. The corrected Figure 5A appears below.

**FIGURE 5:**
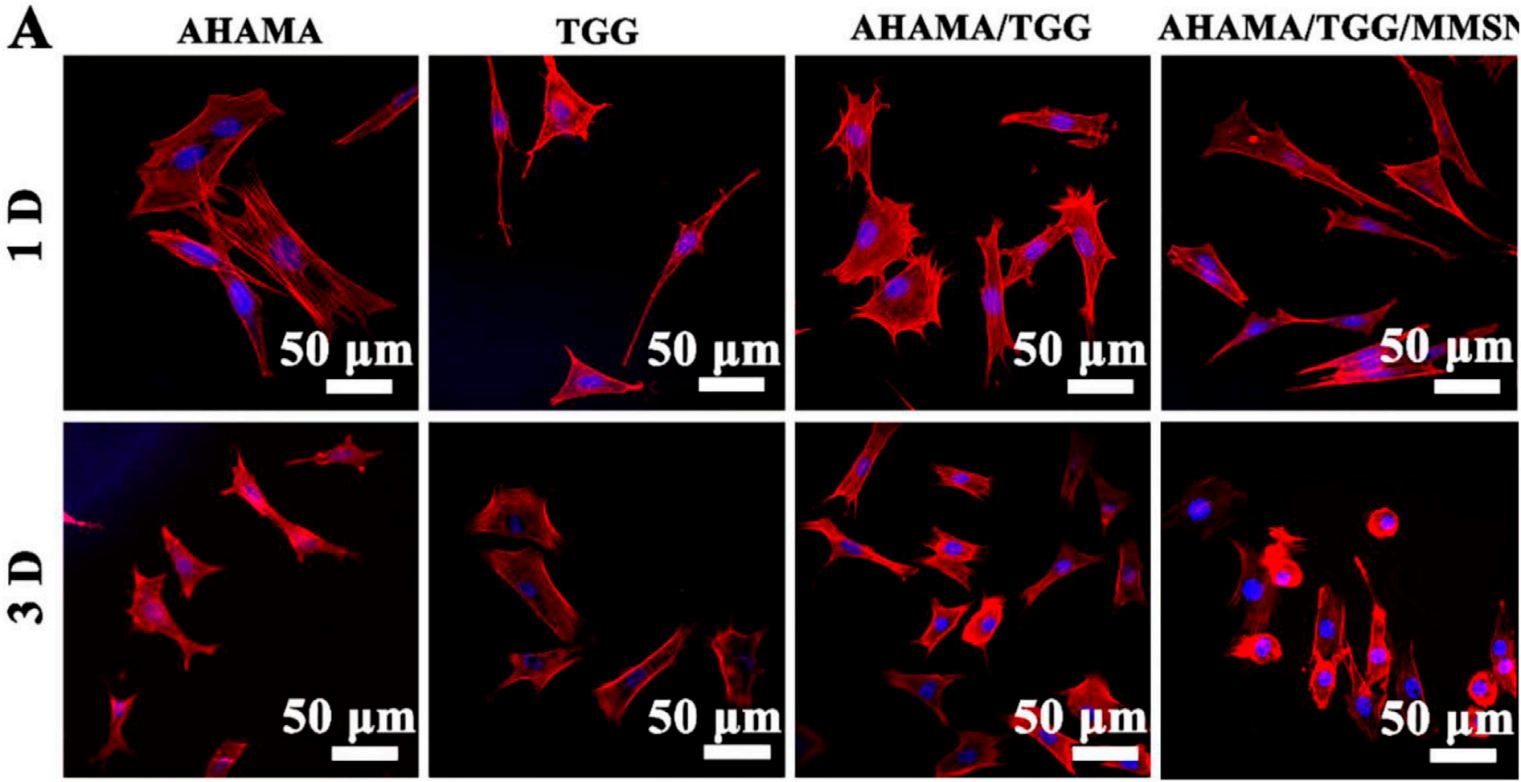
**(A)** CLSM images of stained BMSCs cells showing morphology adhered on the 3D printing AHAMA, TGG, AHAMA/TGG and AHAMA/TGG/MMSN scaffolds on day 1 and 3, respectively.

The original version of this article has been updated.

